# Complete Genomic and Lysis-Cassette Characterization of the Novel Phage, KBNP1315, which Infects Avian Pathogenic *Escherichia coli* (APEC)

**DOI:** 10.1371/journal.pone.0142504

**Published:** 2015-11-10

**Authors:** Jung Seok Lee, Ho Bin Jang, Ki Sei Kim, Tae Hwan Kim, Se Pyeong Im, Si Won Kim, Jassy Mary S. Lazarte, Jae Sung Kim, Tae Sung Jung

**Affiliations:** 1 Laboratory of Aquatic Animal Diseases, Institute of Animal Medicine, College of Veterinary Medicine, Gyeongsang National University, Jinju, 660-701, South Korea; 2 Laboratory of Gene Technology, KU Leuven, Kasteelpark Arenberg 21, 3001, Leuven, Belgium; 3 KBNP Technology Institute, KBNP, Inc., Yesan, Choongcheongnam-do, South Korea; Swinburne University of Technology, AUSTRALIA

## Abstract

Avian pathogenic *Escherichia coli* (APEC) is a major pathogen that causes avian colibacillosis and is associated with severe economic losses in the chicken-farming industry. Here, bacteriophage KBNP1315, infecting APEC strain KBP1315, was genomically and functionally characterized. The evolutionary relationships of KBNP1315 were analyzed at the genomic level using gene (protein)-sharing networks, the Markov clustering (MCL) algorithm, and comparative genomics. Our network analysis showed that KBNP1315 was connected to 30 members of the *Autographivirinae* subfamily, which comprises the SP6-, T7-, P60-, phiKMV-, GAP227- and KP34-related groups. Network decomposition suggested that KBNP1315 belongs to the SP6-like phages, but our comparison of putative encoded proteins revealed that key proteins of KBNP1315, including the tail spike protein and endolysin, had relative low levels of amino acid sequence similarity with other members of the SP6-like phages. Thus KBNP1315 may only be distantly related to the SP6-like phages, and (based on the difference in endolysin) its lysis mechanism may differ from theirs. To characterize the lytic functions of the holin and endolysin proteins from KBNP1315, we expressed these proteins individually or simultaneously in *E*. *coli* BL21 (DE3) competent cell. Interestingly, the expressed endolysin was secreted into the periplasm and caused a high degree of host cell lysis that was dose-dependently delayed/blocked by NaN_3_-mediated inhibition of the SecA pathway. The expressed holin triggered only a moderate inhibition of cell growth, whereas coexpression of holin and endolysin enhanced the lytic effect of endolysin. Together, these results revealed that KBNP1315 appears to use a pin-holin/signal-arrest-release (SAR) endolysin pathway to trigger host cell lysis.

## Introduction

Avian colibacillosis, which is caused by strains of avian pathogenic *Escherichia coli* (APEC), is a severe bird disease that affects the poultry industry and is responsible for enormous economic losses worldwide [1, 2]. *Escherichia coli* is typically a commensal microorganism in the intestinal tract of chickens, but APEC strains are extraintestinal pathogenic *E*. *coli* (ExPEC) whose infection is characterized by various symptoms, such as airsacculitis, polyserositis, enteritis, cellulitis, salpingitis, omphilitis, coligranuloma, egg peritonitis and septicemia [1–3]. Other ExPEC strains have been isolated from human urinary tract infections (UTIs) and neonatal meningitis [2, 4, 5]. In addition, the antibiotic treatment capable of controlling this pathogen has been imprudently used, leading to the appearance of multi-antibiotic resistant APEC stains [6–9]. Therefore, we urgently need an alternative agent or strategy for treating antibiotic-resistant *E*. *coli*.

Bacteriophages (or phages), predators of bacteria, are the most abundant organisms on the planet and contribute to the reservoir of genetic diversity in the microbial environment [10, 11]. Recently, phage genomics have been advancing as a result of the remarkable development in phage genome sequencing, which contributes to the progress of genetic, biotechnological and clinical tools [12, 13]. However, because the process of phage evolution is being affected by frequent horizontal gene transfer (HGT), phage genomes show pervasive mosaicism and it is difficult to characterize them by classical taxonomic study based on hierarchical classification [10, 11, 13]. To overcome the limitation of the classic approach, Lima-Mendez *et al*. [11] proposed a reticulated network model representing the phage-phage relationship in consideration of shared gene (protein) contents, including the evolutionary reconstructions of individual gene phylogeny; this model gives a clear understanding of the true evolutionary history of phage genomes [10, 14].

Bacteriophage SP6 is a well-characterized member of the *Autographivirinae* subfamily (which infects *Salmonella enterica* Typhimurium LT2), which is closely related to the *Escherichia coli* phages, K1-5, K5 and K1E [15]. Collectively, these phages were traditionally considered very similar to the T7-like phages. However, Scholl *et al*. proposed the novel notion that these phages are separate from the T7 group, and instead form a novel genus called the “SP6-like phages” [16]. Several proteins of these phages have been functionally characterized, including those related to phage replication (RNA polymerase) and host specificity determination (tail spike) [17, 18]. Although the phages of this group have been studied in detail, only a single functional study has examined the host lysis proteins of a putative member of the SP6 group: the *Erwinia amylovora* phage, Era103 [19]. To better understand the host lysis mechanisms of this group, further functional studies of lytic proteins are needed [19].

At the final stage of their life cycle, the double-stranded DNA bacteriophages generally use two proteins, holin and endolysin, to lyse the host bacterium by degrading the inner membrane and peptidoglycan layer of the bacterial membrane, respectively [20, 21, 22]. This canonical mechanism was traditionally considered to be a universal strategy through which phages escape from their host bacteria [23]. However, other lytic mechanisms have since been reported, including that involving pin-holin and SAR (signal-anchor-release) endolysin, which are seen in phages P1, 21, phiKMV, and JG068. The endolysins of these phages possess N-terminal SAR domains that are exported to the periplasm via the host *sec* system by virtue of an N-terminal signal sequence. There, the positively charged anchors of the SAR domain tether the exported endolysin to the cytoplasmic membrane in an inactive form. Pin-holin (which differs from canonical holin) then forms a small hole that induces membrane depolarization and releases the tethered endolysin into the periplasm in an active form [24–27]. Similarly, the endolysin of *Oenococcus oeni* bacteriophage fOg44 has a typical signal sequence with the peptidase recognition site, and it is activated by the proteolytic cleavage of the N-terminal signal peptide [28].

Here, we report a genomic analysis of the novel APEC phage, KBNP1315, and the functional characterization of its lysis-cassette. To understand the evolution of this phage, we used gene (protein)-sharing network and comparative genomic analyses to assess its relationships with its relatives. In addition, we characterized the lytic proteins, holin and endolysin, of KBNP1315 to provide novel insights into the host lysis strategy of the SP6-like phages.

## Materials and Methods

### Bacterial strain and characterization

An *E*. *coli* strain was isolated from a chicken farm located in Yesan, South Korea. Phages were isolated from sewage samples obtained from the same chicken farm from which the host bacterium was isolated. The isolated bacteria was identified by using MALDI-TOF MS (Bruker Daltonik GmbH, Leipzig, Germany) and named “KBP1315.” To determine its pathotype, the APEC-specific genes, fimC, tsh, ironN, fyuA, irp2, and stx2, were detected by PCR. In addition, a disk diffusion method was used to evaluate the antimicrobial susceptibility of *E*. *coli* KBP1315 on LB agar plates [28].

### Bacteriophage isolation

Phages were isolated from sewage samples obtained from the same chicken farm from which the host bacterium was isolated. Briefly, each sewage sample was centrifuged at 5,000×g for 10 min, and then filtered through a 0.22 membrane (Merk Millipore, MA, USA). The phage solution was cultured with *E*. *coli* KBP1315 and a plaque assay was performed [29]. After incubation for 24 h at 30°C, a single plaque was picked from plaque assay, eluted in LB broth and combined with a host culture again. Following several rounds of plaque assay and single plaque selection, bacteriophage KBNP1315 was isolated. To purify phage KBNP1315, the phage particles were propagated up to 200 ml and centrifuged at 3,000×g for 30 min at 4°C. The supernatant was carefully collected, treated with 10% polyethylene glycol (PEG) for 1 hr, and centrifuged at 16,000×g for 15 min at 4°C. The precipitate was resuspended in TM buffer (10 mM Tris-HCL, 100 mM NaCl, 10 mM MgCl_2_, and 10 mM CaCl_2_, pH 7.5) and purified by CsCl density gradient equilibrium centrifugation, as previously described [30].

### Transmission electron microscopy (TEM)

Morphological analysis of purified phage KBNP1315 was performed using TEM, as previously described by Jang *et al*. [10]. Purified phage solution (8 μl) was dropped onto 400-mesh Formvar carbon-coated copper grids (Ted Pella Inc., CA, USA). After 2 min, the grids were stained with 8 μl of 2% aqueous uranyl acetate (Merck, Darmstadt, Germany) and examined with a Philips TECNAI F12 FEI transmission electron microscope (FEI, OR, USA) at an accelerating voltage of 120 kV. KBNP1315 was morphologically classified according to the classification scheme of the International Committee on Taxonomy of Viruses (ICTV; http://www.ictvdb.org/).

### Genomic sequencing and bioinformatic analysis

KBNP1315 DNA was prepared for sequencing as previously described [31], using proteinase K digestion followed by phenol-chloroform-isoamyl alcohol extraction and ethanol precipitation. The genomic sequence of KBNP1315 was obtained using an FLX Titanium genome sequencer (Roche, Mannheim, Germany) according to the manufacturer's standard procedures. All reads were assembled with Newbler Assembler, version 2.3 (454 Life Sciences, Branford, CT). Sequencing and assembly were performed by ChunLab Inc. (Seoul, South Korea), and open reading frames (ORFs) were predicted and annotated using Rapid Annotation Subsystem Technology, version 2.0 (RAST; http://rast.nmpdr.org). The potential gene products of the ORFs were compared with the proteins in the nonredundant GenBank databases using BLASTp or PSI-BLAST (http://www.ncbi.nlm.nih.gov/blast/) [32], with an E values of < 10^-4^. The functional assignment of each ORF was predicted using HHpred, version 2.0 (http://toolkit.tuebingen.mpg.de/hhpred) [33]. To search for tRNA genes, we used tRNAscan-SE, version 1.2.1 (http://lowelab.ucsc.edu/tRNAscan-SE/) [34]. The PHIRE program, version 1.00 (http://www.agr.kuleuven.ac.be/logt/PHIRE.htm) [35], was used to identify phage-specific promoters under the following default parameters: string length, 20; degeneracy, 4; dominanNum, 4; and window size, 20. The ARNold program (http://rna.igmors.u-psud.fr/toolbox/arnold/) [36] was used to predict rho-independent transcription terminators.

### Protein family clustering

To cluster the protein sequences predicted from the KBNP1315 genome (n = 62) into orthologous families, we performed pairwise similarity comparisons using BLAST in the A CLAssification of Mobile genetic Elements database, version 0.4 (http://aclame.ulb.ac.be), using an E-value cutoff of 0.0001 [37]. Thirty-four potential gene products of KBNP1315 were found to be associated with protein families in the “viruses” database (as of Jan, 2015), while the remaining proteins were assigned to uncharacterized protein families. The unassigned protein sequences were compared with the protein sequences in the HMMER database (E < 10^-4^) [38]. Because the ACLAME database lacked information on recently-identified phages [10], we further directly examined protein families from the following putative relatives of KBNP1315: SP6 (GenBank accession number AY370673), K1E (AM084415), K1-5 (NC_008152), ACG-c91 (NC_019403), Era103 (NC_09014), phiEa1H (FQ482084), phiEa100 (NC_019926), PP1 (NC_019542), Vc1 (KJ502657), T7 (GU071091), T3 (NC_003298), YeO3-12 (NC_001271), phiA1122 (NC_004777), gh-1 (NC_004665), Berlin (NC_008694), K1F (NC_007456), phiKMV (NC_005045), VP4 (NC_007149), P60 (NC_003390), P-SSP7 (NC_006882), Syn5 (NC_009531), LKA1 (NC_009936), LKD16 (NC_009935), KP34 (NC_013649), F19 (NC_023567), VP93 (NC_012662), GAP227 (NC_020078), phiAS7 (NC_019528), and phi80-18 (NC_019911). A subset of predicted protein sequences (n = 564) retrieved from ACG-c91, phiEa1H, phiEa100, PP1, Vc1, KP34, F19, VP93, GAP227, phiAS7, and phi80-18 were subjected to clustering by comparison with the ACLAME and HMMER databases, as described for KBNP1315.

### Network construction, analysis, and decomposition into clusters

The results of pairwise comparisons were used to generate a matrix in which the rows represented mobile genetic elements (MGEs) and the columns represented the protein families. We then determined the similarities of KBNP1315, ACG-c91, PP1, phiEa1H, phiEa100, VC1, KP34, F19, VP93, GAP227, phiAS7, and phi80-18 to their respective related MGEs, as previously described [11]. We determined the phage-phage similarity (the Sig score) as the minus logarithmic score by multiplying the hypergeometric similarity P-value by the total number of pairwise comparisons [11].

MGEs with Sig values > 1 were used to construct a new network of bacteriophage genome-encoded protein-sharing relationships among KBNP1315 and its potential relatives. The network was visualized with the Cytoscape software, version 3.0.2 (http://cytoscape.org/) [39], which uses an edge-weighted spring-embedded model in which MGEs that share more protein families are placed closer together in the network. We then estimated the topological properties of the network with the Network Analyzer 2.7 Cytoscape plug-in [11].

The Markov clustering (MCL) algorithm was used to assign phages into clusters. The main parameter of MCL algorithm is called “inflation”, which influences the number and size of the clusters. We tested all inflation values from 1.2 to 5 by steps of 0.2 and analyzed each clustering result to maximize the cluster homogeneity. We then calculated the membership as the proportion of edges (or the “weight”) that linked each node to each cluster, using the Network Analysis Tools (NeAt; http://rsat.ulb.ac.be/neat/) [40].

### Functional characterization of the lysis cassette of KBNP1315

The primers and plasmids used throughout this study are listed in S1 Table. To construct plasmids encoding the putative holin and endolysin genes of KBNP1315, the phage DNA was PCR amplified with specific primers hol F and -R, and endolysin F and -R. The obtained PCR products were cloned into the pET-DEST42 expression vector (Invitrogen, CA, USA) to generate pDEST-hol and pDEST-lys. To clone holin and endolysin together, pDEST-hol, including the T7 promoter and terminator, was amplified with primers insert F and -R. The pDEST-lys was linearized by PCR using primers linearizing F and -R, which contained 16 bp of overlapping homologous sequences found in the amplified fragment of pDEST-hol. The PCR products were purified and fused to form a circular plasmid containing both ORFs, using an EZ-fusion cloning kit (Enzynomics, Dajeon, Korea). All constructs were verified by DNA sequencing analysis. The generated plasmids were transformed to *E*. *coli* BL21 (DE3), and expression was induced with 0.3 or 1 mM IPTG. To block the SecA secretion system, 0.2, 1, or 5 mM sodium azide (NaN_3_) was added at the time of induction. For our growth pattern analysis, the optical density was measured by spectrometry at 600 nm every 30 minutes or 1 hr.

### Nucleotide sequence accession numbers

The sequences of bacteriophage KBNP1315 have been deposited in GenBank under accession number KJ749827.

## Results

### Determination of *E*. *coli* KBP1315 as an APEC strain

To determine whether *E*. *coli* strain KBP1315 represented an APEC strain, we performed PCR amplification of six well-characterized virulence genes [41–43]: hlyF (hemolysin), papC (p-fimbria), iucD, fyuA, irp2, and iroN (iron uptake-related gene). All six APEC-virulence genes were identified in this bacterium (S1 Fig).

### Phage morphology

**Fig 1:**
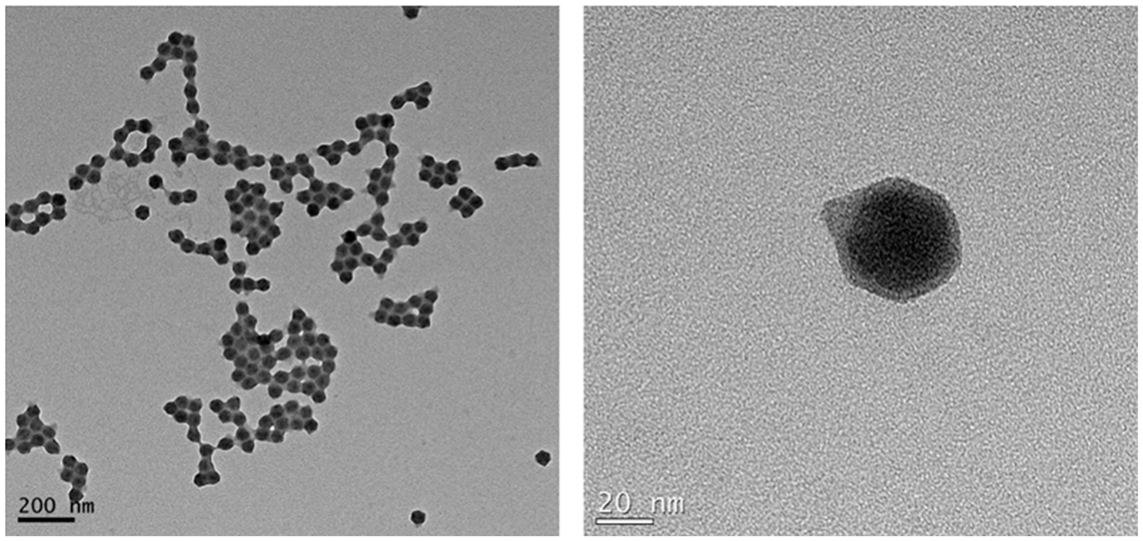
Morphology of bacteriophage KBNP1315. Transmission electron micrograph (TEM) of purified phage particles negatively stained with 2% uranyl acetate.

TEM-based showed that phage KBNP1315 has an icosahedral capsid and a short tail (Fig 1). The phage head was 47.71 ± 1.51 nm in diameter, while its tail was 11.9 ± 1.64 nm in length. Based on these morphological characteristics, we propose that KBNP1315 belongs to the C1 morphotype of the *Podoviridae* family [44].

### Genome sequencing and annotation

The genome of KBNP1315 is a linear dsDNA of 45,403 bp, including 208 bp of direct terminal repeats (GC content, 45.89%). Sixty-two open reading frames (ORFs) were predicted using an automatic analysis tool available through the RAST server. Annotation of the predicted proteins by BLASTp, PSI-BLAST, and HHpred identified only 27 of the ORFs. They were categorized into the following functional groups: a) transcription/translation (ORF 6, ORF 9, and ORF 21); b) DNA replication/modification (ORF 13, ORF 22, ORF 26, ORF 27, ORF 28, ORF 29, ORF 31); c) phage morphogenesis (ORF 37, ORF 38, ORF 39, ORF 40, ORF 41, ORF 44, ORF 45, ORF 47, ORF 48, ORF 49, ORF 56); d) host lysis (ORF 51); and e) nucleotide metabolism (ORF 11, ORF 20, ORF 30, ORF 35, and ORF 50). Seven phage-specific promoters were predicted, all of which were predicted to function in the rightward direction. We identified three rho-independent transcription terminators. No tRNA motif was predicted (S2 Table).

### Pairwise comparison of KBNP1315

Pairwise comparison of the predicted ORFs of KBNP1315 and its relatives (SP6, Vc1, K1E, K1-5, ACG-c91, Era103, phiEa1H, Ea100, PP1, T7, T3, YeO3-12, gh-1, K1F, Berlin, phiA1122, VP4, P60, Syn5, P-SSP7, phiAS7, GAP227, phi80-18, phiKMV, LKA1, LKD16, KP34, F19 and VP93) against 11,475 protein families from 457 virus (phage) genomes allowed us to classify 1,366 protein sequences into 531 protein families, including 178 uncharacterized protein families (S3 Table) [45]. The resulting matrix (in which columns represented protein families and rows represented phage genomes) revealed that KBNP1315 shared the most protein families with phages belonging to the *Autographivirinae* subfamily, especially the SP6- and T7-like phages [46]. Among these phage groups, we identified seven protein families common to the genomes of KBNP1315 and its relatives (more than 29 phages), namely families vir:14 (putative DNA polymerase function), f:v:81 (terminase large subunit), f:v:99 (portal protein), f:v:100 (exonuclease), f:v:120 (RNA polymerase) and f:v:133 and 134 (tail fibers). Our findings suggest that these sets of genetic components appear to have been conserved during the evolution of the *Autographivirinae* phages. Notably, RNA polymerase was previously reported as signature for the *Autographivirinae* phages [47].

### Network construction, analysis and decomposition

**Fig 2:**
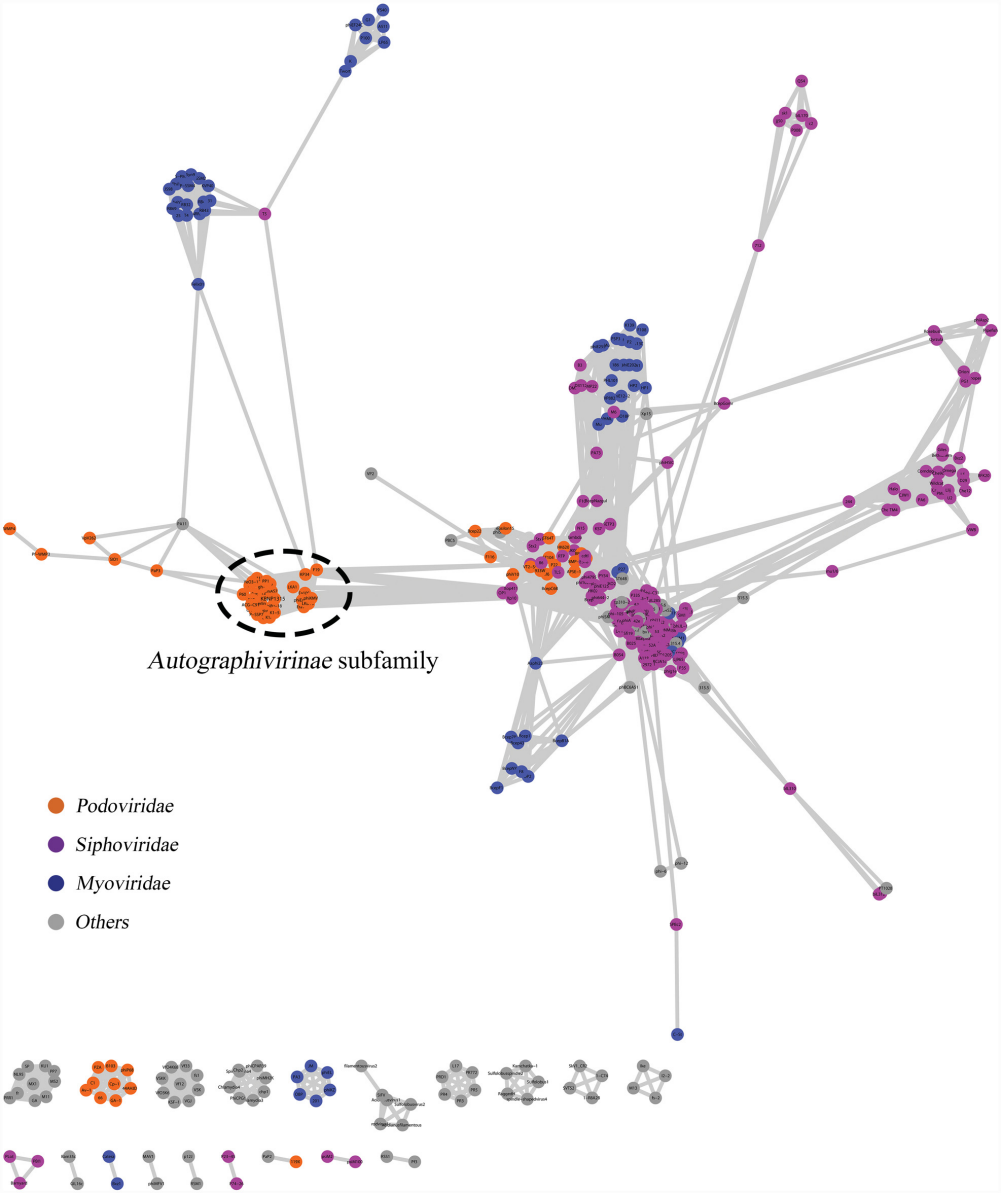
Protein-sharing network of KBNP1315. A network for KBNP1315 was produced using Cytoscape 3.1.2. Nodes represent phages, while edges indicate significant relationships between nodes. Each node is colored in accordance with its ICTV classification.

Based on the number of protein families shared between the considered phages, we established a weighted network in which each node represented the phage and each edge represented the phage-phage similarity (called the Sig score) [10, 11]. To analyze the relationships among the nodes, we computed network topological values, including the clustering coefficient (CC, which represents the interconnectivity scale of a given node with the neighbors) and the betweenness centrality (BC, which indicates the extent which a given node lies on the shortest path between all the other nodes) [48]. The resulting network consisted of 431 nodes belonging to *Podoviridae*, *Myoviridae*, *Siphoviridae*, and uncharacterized/other phages; 6,823 edges; and 20 networks of different sizes (Fig 2). Notably, KBNP1315 was located in the peripheral region of the largest network that comprised a dense cluster of phages belonging to the *Autographivirinae* subfamily (SP6-, T7-, phiKMV-, and P60-) and putative members of this subfamily (GAP227, phiAS7, phi80-18, KP34, F19, and VP93) [46, 47, 49]. In terms of network topologies, the phages, KBNP1315 and its relatives formed a highly interconnected cluster with an average CC value of 0.94 (absolute cohesiveness = 1) (data not shown). Among the highly interconnected members of *Autographivirinae*, some nodes (e.g., those representing phiKMV, LKD16, LKA1, phiEa1H, Ea100, Era103, KP34, and F19) were slightly closer to the center of network, perhaps reflecting that these phages had multiple connections with other phages.

**Fig 3:**
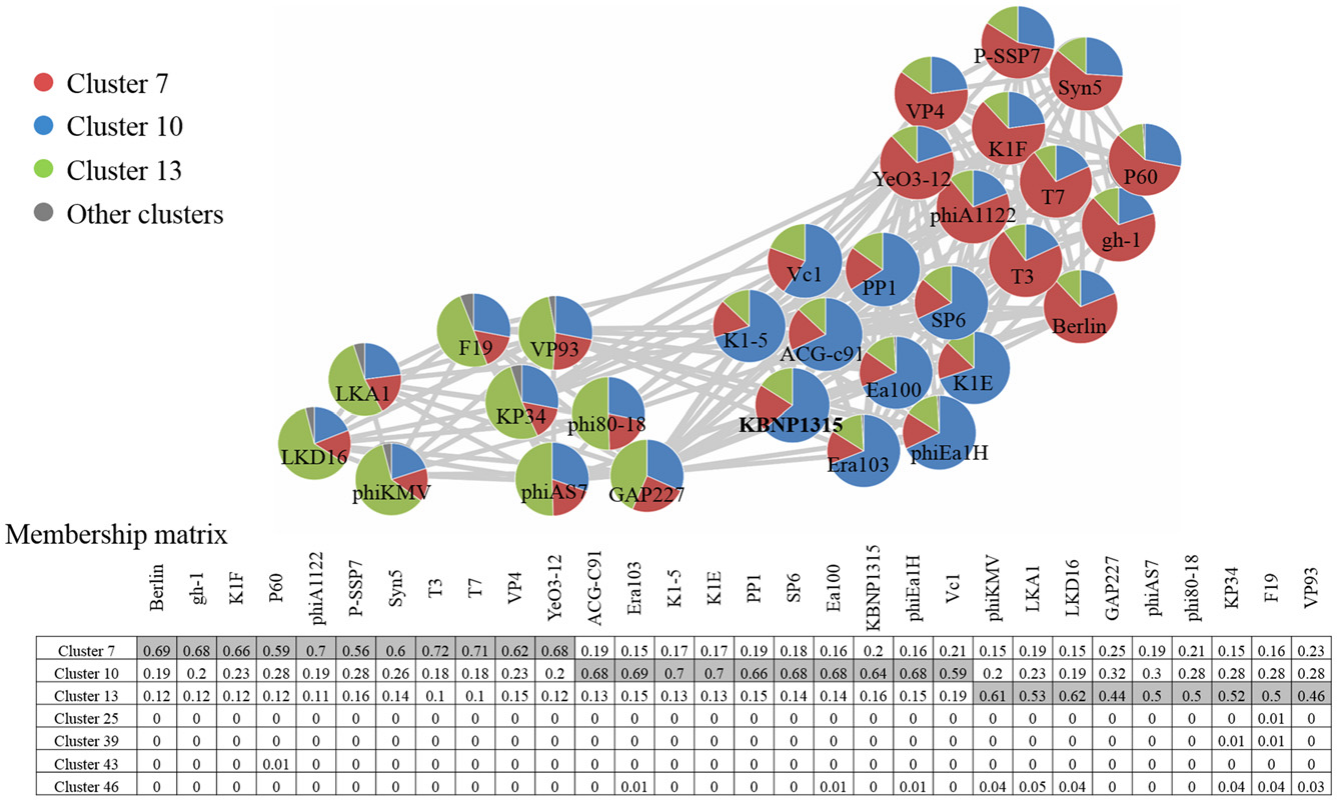
*Autographivirinae* subfamily phages are represented as an independent network. Each node is illustrated as a pie chart, with the wedges representing the proportion of edges connected with nodes belonging to each cluster. The left panel shows the clusters in different colors. The lower panel represents the membership matrix. The rows indicate phages related to *Autographivirinae* subfamily and the columns represent the clusters that share wedges with the nodes more than 0.01. Shared boxes indicate the clusters into which the phages were assigned by the MCL algorithm.

The MCL algorithm was used to clarify subgroups of the *Autographivirinae* phages as this has been a useful approach for extracting groups of densely connected phage genomes (i.e., clusters) from such networks [40, 50]. As a result, MCL clustering with an inflation value of 2.6 (data not shown) produced a total of 64 clusters (S4 Table). As a result, the 30 phages of the *Autographivirinae* group including KBNP1315 were classified into clusters 7, 10, and 13, comprising SP6-like phages, T7-like phages plus three unassigned phages (P60, Syn5, and P-SSP7) and phiKMV-like phages plus KP34 like phages, respectively. We further analyzed the membership of each node within these three clusters to represent how strongly each node is related to each cluster [11, 40]. The obtained membership matrix is represented by an independent network and the included phages all showed the highest membership to their respective clusters, supporting that these phages were appropriately clustered by MCL algorithm (Fig 3). Interestingly, KBNP1315 exhibited a much higher membership to cluster 10 (0.64) compared to cluster 7 (0.2) or 13 (0.16), indicating that it has a strong relationship with the SP6-like phages. On the other hand, the 11 phage members of cluster 7 showed overall high memberships to cluster 7 (0.68 on average), with the exceptions of P60, Syn5, and P-SSP7 (less than or equal to 0.6), suggesting that the latter three phages have a more distant relationship with the T7-like phages. Also, nine phages including phiKMV-, KP34- and GAP227-like phages classified into cluster 13 revealed high degrees of mosaicism and chimerism, reflecting that they shared complicated relationships with other members of the network (Fig 2). In particular, KP34 and F19 were assigned to five and six clusters, respectively; this is consistent with their high BC value and provides additional evidence that they may function as bridges between unrelated phages.

### Comparative genome analysis of KBNP1315

**Fig 4:**
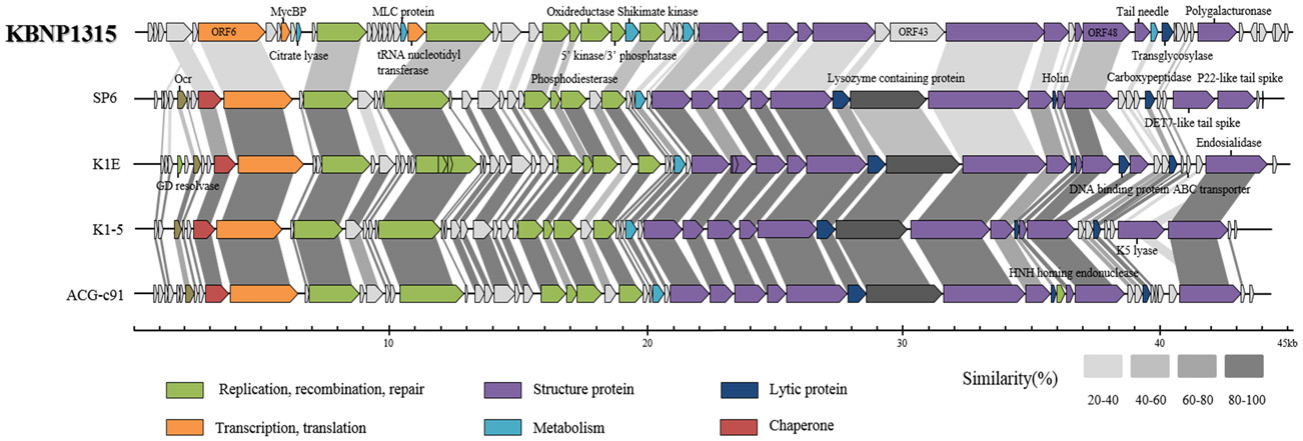
Comparative genome analysis among KBNP1315 and select relatives (KBNP1315, SP6, K1E, K1-5, and ACG-c91). Each ORF is represented by a color assigned according to the protein function. The degree of amino acid similarity between paired ORFs was analyzed by BLASTp search, and the percentage of similarity is represented in color from pale gray to dark gray.

To better understand the evolutionary relationship between KBNP1315 and the SP6-like phages, the genome of KBNP1315 was compared with its closest relative genomes (SP6, K1E, K1-5, and ACG-c91). As shown in Fig 4, the genome of KBNP1315 was found to have a gene order similar to those of the other SP6-like phages, but its ORFs had relatively low sequence identities with those of the other SP6-like phages: 23 to 58% with SP6 (average, 41%) and 25 to 64% with K1E (40%) (Fig 4 and S5 Table). Interestingly, our comparison of SP6 phage that infects a different host (*Salmonella enterica* Typhimurium LT2) versus several coliphages (K1E, K1-5, and ACG-c91) revealed higher average similarities (74, 72, and 71%, respectively) than those with KBNP1315 (S5 Table). This suggests that KBNP1315 may have shared a common ancestor with the SP6-like phages, but diverged from them some time ago [13, 16].

In addition, comparative analysis revealed a unique feature with respect to the host specificity of KBNP1315. Deduced ORF 56, which is encoded at the far right end of the genome, is a putative tail spike protein that is likely to play an important role in recognizing the host outer membrane. Our HHpred analysis revealed that ORF 56 had strong similarities to the endo-polygalacturonase from *Erwinia carotovora* ssp. carotovora and this enzyme is totally different from those of its relatives (SP6; DET7-liketail spike and P22-like tail spike/ K1E, K1-5, ACG-c91; endosialidase, K5 lyase (only K1-5)).

We further identified the separated position of host cell lysis genes of KBNP1315, including holin, endolysin, Rz and Rz1 (Fig 4). ORFs 46, 51, 54, and 55 of KBNP1315 encode putative versions of holin, endolysin, Rz, and Rz1, respectively. Despite the conserved position of these lysis genes, the putative holin, endolysin, Rz and Rz1 sequences of KBNP1315 share low amino acid similarities with those of the other SP6-like phages, with the putative endolysin-encoding sequence failing to show any similarity. We therefore speculated that KBNP1315 may use a different strategy to lyse the host bacterial cell wall at the end of the infection cycle, compared to the other SP6-like phages. Consistent with this, most phages with known lytic mechanisms have their lysis genes encoded in canonical serial order [24], whereas those of KBNP1315 are encoded separately. To better understand the mechanisms through which KBNP1315 lyses the host cell, we focused on the functions of holin and endolysin, which cleave the inner cell membrane and the peptidoglycan layer, respectively, of the bacterial cell wall.

### Functional analysis of the lysis proteins of KBNP1315

Our analysis with the SignalP 4.1 server revealed that ORF 51 possesses a putative signal sequence and a cleavage site embedded in the C-region, but has no putative positive anchor region at its N-terminus (S2 Fig). Thus, we propose that ORF 51 encodes a putative SAR endolysin that should be secreted in a membrane-tethered form via the host *sec* system and be released via signal peptidase cleavage [23].

**Fig 5:**
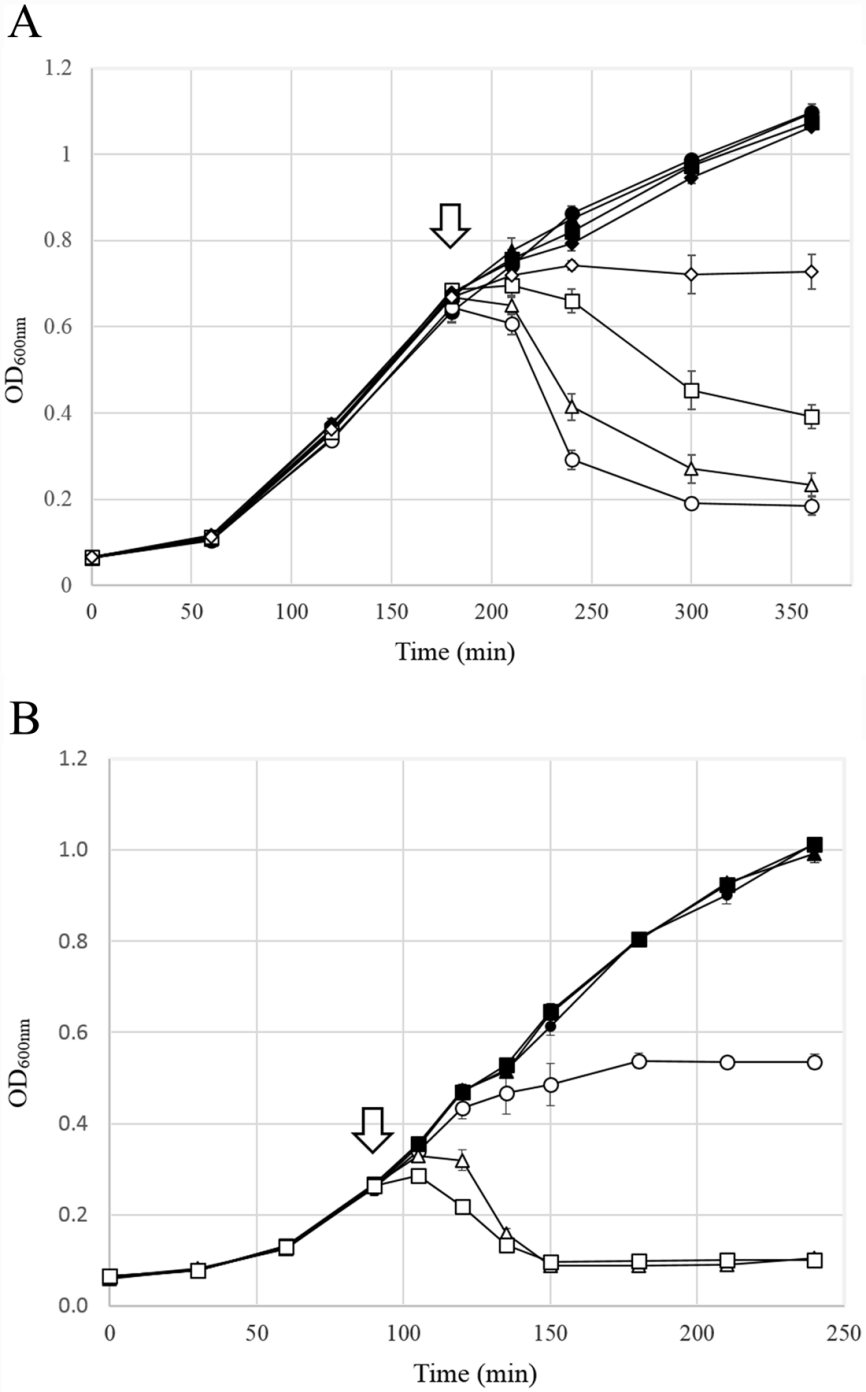
Growth curves of *E*.*coli* BL21 cells expressing two putative lysis proteins of KBNP1315. (A) ORF 51 (putative endolysin) was expressed in *E*.*coli* BL21 in the presence of different concentrations of NaN_3_. IPTG (1 mM) was added at 90 min (arrow) in the induction group (open symbols). The non-induction group is represented with closed symbols. NaN_3_ was simultaneously applied at concentrations of 0 mM (circle), 0.1 mM (triangle), 0.5 mM (square), and 1 mM (diamond). (B) Growth curves of induction (open symbols) and non-induction (closed symbols) groups expressing ORF46/pDEST42 (circle), ORF51/pDEST42 (triangle), and ORF46/ORF51/pDEST42 (square) are shown. Induction was performed by the addition of 0.3 mM IPTG (arrow).

To evaluate the involvement of the host *sec* system in the exportation of the putative SAR endolysin of KNBP1315, we induced the expression of this endolysin in *E*.*coli* BL21 (DE3) competent cells using 1 mM IPTG, added sodium azide (NaN_3_, at 0.1, 0.5, and 1 mM; to inhibit the ATPase activity of SecA) to induced or non-induced cultures, and observed cell growth by monitoring the absorbance at 600 nm. In the NaN_3_-free induction group, the addition of 1 mM IPTG resulted in cell lysis: the optical density decreased slightly from 0.645 to 0.607 over the first 30 minutes after induction, and then decreased rapidly from 0.607 to 0.292 over the next 30 minutes due to rapid cell lysis (Fig 5A). In contrast, the NaN_3_-treated induction groups showed dose-dependent delays in lysis (Fig 5A). The 0.1 mM NaN_3_-treated group showed a lysis pattern similar to that of the NaN_3_-untreated group, although there was a slight post-induction decline in OD. Compared with the untreated control group, in which rapid lysis was observed 30 minutes post-induction, the 0.5 mM NaN_3_-treated group showed a delayed lysis pattern, with lysis first observed at 30 minutes post-induction and drastically increased (as reflected by a sharp decrease in OD) over the succeeding 30 minutes. The group treated with 1 mM NaN_3_ showed a complete blockade of cell lysis for at least 3 hours post-induction, and did not have any noticeable effect on non-induced cells. Based on these results, we conclude that KBNP1315 ORF51 acts as a typical SAR endolysin [24] by causing host cell lysis via the host *sec* pathway.

Next, we analyzed the function of the putative holin protein in KBNP1315. The deduced amino acid sequence of ORF 46 was predicted to possess a single transmembrane domain (residues 35 to 57) and a putative N-out, C-in topology, suggesting that the gene product of ORF 46 has a structure similar to that of the class III holins (S3 Fig) [51]. To characterize the holin-like protein of KBNP1315, we cloned ORF 46 and/or ORF 51 into the pDEST42 vector and transformed the resulting plasmids to *E*. *coli* BL21. To examine the lytic function of the putative holin protein, we gradually expressed ORF 46 with and without ORF 51 using a low concentration of IPTG (0.3 mM). The expression of ORF 46 alone resulted in a moderate decrease of optical density at two hours post-induction (OD = 0.563) (Fig 5B). In contrast, the expression of the putative endolysin (ORF 51) alone yielded a dramatic decrease in optical density at 90 min post-induction (OD = 0.089). The coexpression of both ORF 46 and ORF 51 was associated with an immediate response (a slight decrease in growth) following the addition of IPTG. There was no difference in the time taken to achieve complete lysis between the endolysin- and holin/endolysin-expressing groups; however, the optical density of the coexpression group was more strongly decreased at 60 minutes post-induction (OD_ORF46,51_ = 0.219, OD_ORF51_ = 0.320). Although the difference between the induced and non-induced conditions in terms of optical density after 60 min was greater for the group treated with the higher concentration of IPTG (1 mM) compared to the 0.3 mM induction condition, the two induction conditions yielded similar patterns (data not shown). These observations suggest that holin may not be an essential protein for host lysis in KBNP1315, but rather it appears to work synergistically with endolysin to enhance host lysis.

## Discussion

In this study, we newly purified a coliphage and designated it KBNP1315, as it was shown to infect avian pathogenic *E*. *coli* strain KBP1315. We then performed genomic and functional characterizations of this new phage. Pairwise comparison and a mathematically modeled gene (protein)-sharing network revealed that KBNP1315 has several informative evolutionary connections with phages belonging to the *Autographivirinae* subfamily. Interestingly, the examined phage genomes showed strong conservation of essential genes involved in DNA replication, transcription, packaging and morphogenesis, including those encoding putative RNA polymerase, DNA polymerase, portal protein, terminase large subunit, exonuclease and tail fiber proteins (S3 Table). This allowed the phages to densely connect with one another and form a highly interconnected cluster in the generated protein-sharing network. These observations suggest that the conserved homologs have undergone flow among the studied phage genomes beyond the genus level. Thus, our findings suggest that these phages have a common ancestor and have taken advantage of their shared genetic pool to exchange functional genetic elements during their evolution [52].

Further analysis of the assignment of various nodes to different clusters on the basis of MCL clustering allowed us to classify the members of the *Autographivirinae* subfamily into three subgroups. Especially, KBNP1315 showed its highest membership to cluster 11 which included members of the SP6-like phages. Since the highest value of ICCC helps in identifying clusters of densely related phages consisting of unique genetic module-shared descendants, this result reveals that KBNP1315 is one of the member of SP6-like phages and most likely shares vertically inherited modules with the SP6-like phages [11]. However, although MCL clustering showed a close relationship among KBNP1315 and SP6-like phages, comparative genomic analysis provided three differences in this phage’s genome from those of other SP6-like phages studied herein. First, although KBNP1315 shares a conserved gene order with other members in this group, the amino acid identities are low, possibly indicating that KBNP1315 is the most widely divergent member of the SP6-like phages [16]. Second, KBNP1315 encodes a unique tail spike protein for attachment to the host membrane. As tail spikes give phages their infectious specificity, the observation that the tail spike of KBNP1315 (endo-polygalacturonase) differs from that of other coli phages (e.g., endosialiase, K5 lyase) within the SP6-like phages supports the notion that the former has developed a disparate strategy for host recognition [18, 53, 54]. Third, the holin, endolysin and Rz/Rz1-like proteins of KBNP1315 also differ strongly from those of the other SP6-like phages, indicating that the former uses a different host lysis mechanism [21, 54, 55]. Collectively, these observations may suggest that KBNP1315 shares a common ancestor with the SP6-like phages but diverged a long time ago, and that KBNP1315 uses atypical strategies for infection and host lysis, and appears to have taken advantage of the HGT process to adjust to various environmental factors.

Our observation that ORF 51 could trigger host cell lysis independent of holin function confirms that KBNP1315 uses a SAR endolysin in its lysis system. Although the catalytic triad (E-8aa-D/C-5aa-T) found in the endolysin sequences from P1, 21, phiKMV, Era103 and T4 is not conserved in ORF 51 (S2 Fig), the enzymatic activity of the latter is comparable with those of SAR endolysins containing these catalytic residues [23, 24, 25]. Most SAR endolysins (roughly 85%) contain the lysozyme-type catalytic domain; however, the lack of conserved catalytic residues in ORF 51 may indicate that it has a different type of catalytic domain. Indeed, our HHpred analysis indicated that, unlike the catalytic domains of P1, 21, phiKMV, Era103 and T4 endolysin, that of ORF51 is related to the soluble lytic transglycosylase (SLT) domain found in *Burkholderia*, *Campylobacter*, *Escherichia*, and *Pseudomonas* phage endolysins [56]. We found that expression of ORF51 triggered host lysis without the initial delay observed from several other SAR endolysins; this may reflect the unique structural features of its SAR domain that appears to lack the positively charged anchor and the signal peptide cleavage site [23, 24, 27, 57]. We therefore conclude that the SAR endolysin of KBNP1315 is easily released from the cytoplasmic membrane by the signal peptidase, and possesses quite a different PG-degrading enzyme from other SAR endolysins. On the other hand, another putative lytic protein, encoded by ORF46, moderately inhibited cell growth and had a synergistic effect on SAR endolysin-mediated lysis of host cells. In addition, the secondary structure of ORF 46 (an N-out, C-in topology with a single TMD-spanning α-helix domain spanning residues 33–57) is similar to that of gp5 of mycobacteriophage Ms6, which forms lesions that are actually too small to translocate the canonical endolysins from phage λ and Ms6 [51]. Although the exact role of ORF 46 remains elusive, we suggest that its protein product functions as a pin-holin. Together, our findings strongly suggest that KBNP1315 uses a pin-holin/SAR endolysin pathway for host lysis. We also provide the first experimental evidence that holin and endolysin can have synergistic lytic activity in SP6-like phages.

In summary, we herein used gene (protein)-sharing network and comparative analyses to perform an evolutionary study of novel phage KBNP1315. Our results strongly suggest that this phage is a distantly related member of SP6-like phages with unique genetic features, tail spike and lytic proteins revealing its atypical strategies for infection and host lysis. Moreover, our functional study of two lytic proteins of KBNP1315 demonstrates their important roles for host lysis and we believe that this work could contribute to the development of a novel antimicrobial approach for controlling avian colibacillosis.

## Supporting Information

S1 Fig PCR amplification for detecting the virulence genes of *E*. *coli* strain KBP1315. (PPTX)

S2 Fig Sequence alignment of the N-terminus of phage KBNP1315, P1, 21, phiKMV, Era103 and T4 endolysin. (PPTX)

S3 Fig Sequence of gene coding holin-like protein of KBNP1315. (PPTX)

S1 Table Oligonucleotide primers and plasmids. (XLSX)

S2 Table Genome annotation of KBNP1315. (XLSX)

S3 Table Protein family comparison matrix. (XLSX)

S4 Table Phage clusters produced by MCL algorithm. (XLSX)

S5 Table Amino acid similarities among ORFs from members of SP6-like phages. (XLSX)
