# Supplementary material for: Complete Genomic and Lysis-Cassette Characterization of the Novel Phage, KBNP1315, which Infects Avian Pathogenic Escherichia coli (APEC)
Source: PLoS One. 2015 Nov 10;10(11):e0142504. doi: 10.1371/journal.pone.0142504 (PMC4640515; doi:10.1371/journal.pone.0142504)
Supplement: S1 Fig — (PPTX) [file pone.0142504.s001.pptx]

## Slide 1
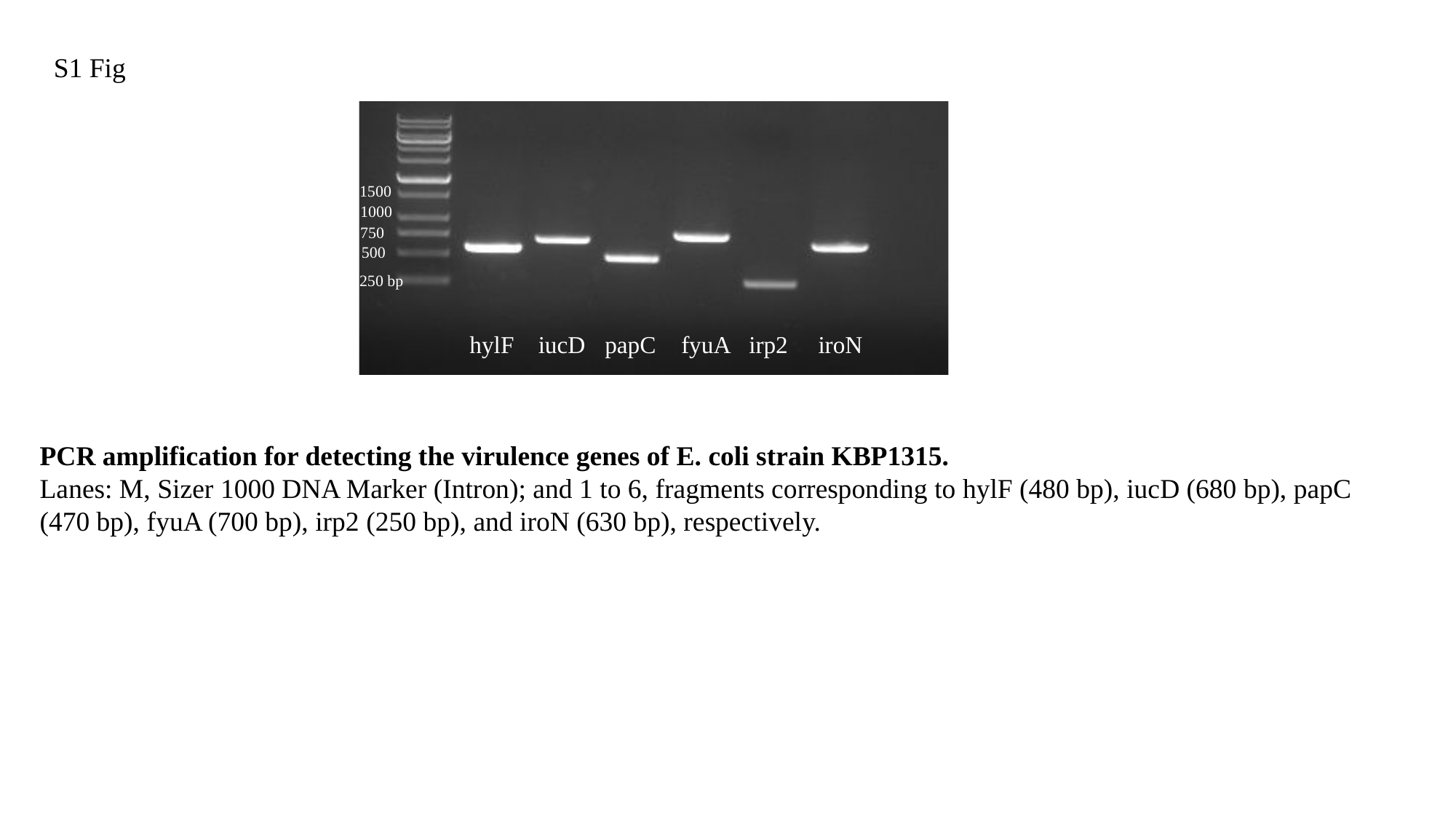

S1 Fig
hylF
iucD
papC
fyuA
irp2
iroN
1500
1000
750
500
250 bp
PCR amplification for detecting the virulence genes of E. coli strain KBP1315.
Lanes: M, Sizer 1000 DNA Marker (Intron); and 1 to 6, fragments corresponding to hylF (480 bp), iucD (680 bp), papC (470 bp), fyuA (700 bp), irp2 (250 bp), and iroN (630 bp), respectively.
