# Supplementary material for: Complete Genomic and Lysis-Cassette Characterization of the Novel Phage, KBNP1315, which Infects Avian Pathogenic Escherichia coli (APEC)
Source: PLoS One. 2015 Nov 10;10(11):e0142504. doi: 10.1371/journal.pone.0142504 (PMC4640515; doi:10.1371/journal.pone.0142504)
Supplement: S2 Fig — (PPTX) [file pone.0142504.s002.pptx]

## Slide 1
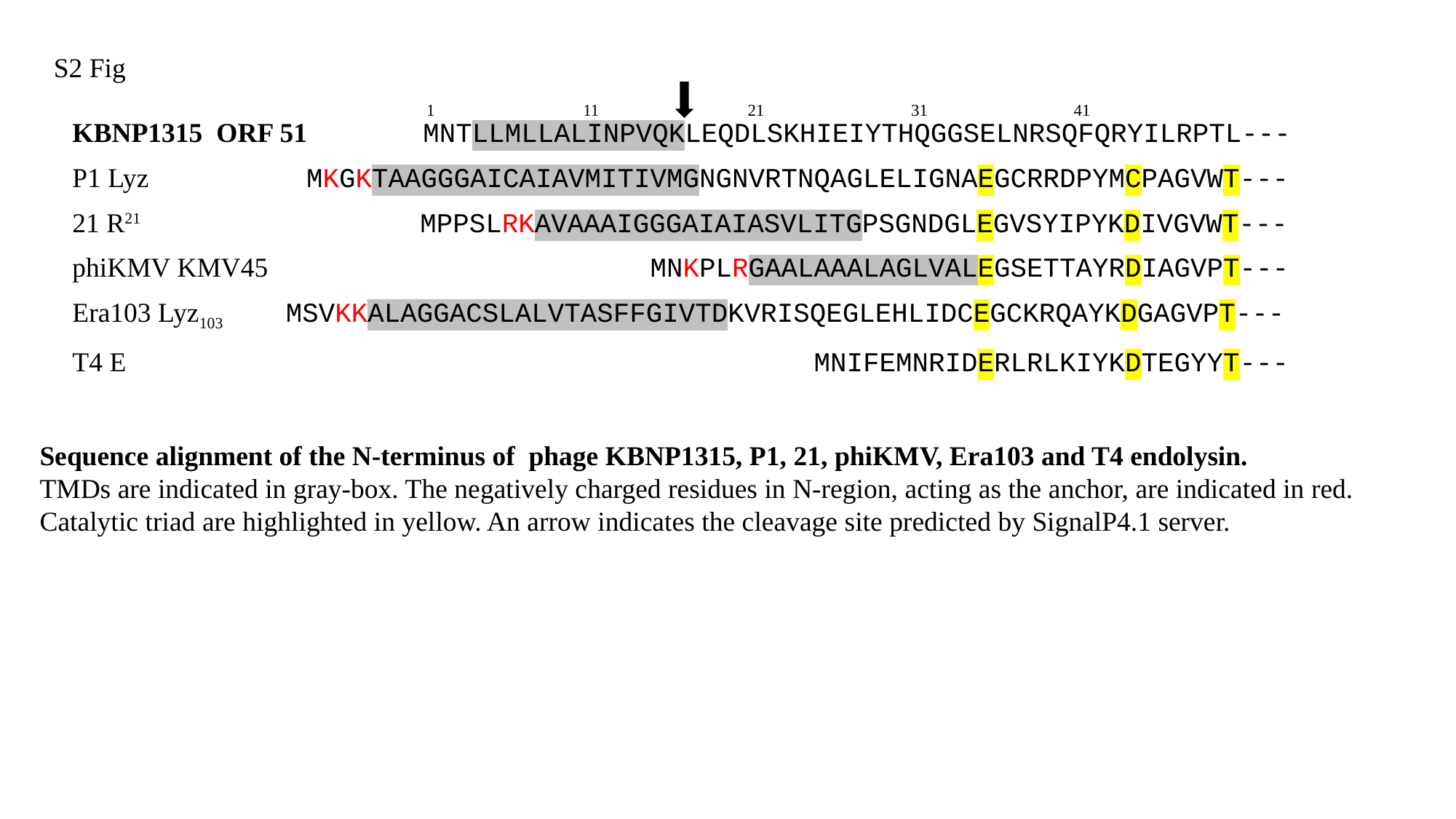

S2 Fig
1
11
21
31
41
KBNP1315 ORF 51 MNTLLMLLALINPVQKLEQDLSKHIEIYTHQGGSELNRSQFQRYILRPTL---
P1 Lyz MKGKTAAGGGAICAIAVMITIVMGNGNVRTNQAGLELIGNAEGCRRDPYMCPAGVWT---
21 R21 MPPSLRKAVAAAIGGGAIAIASVLITGPSGNDGLEGVSYIPYKDIVGVWT---
phiKMV KMV45 MNKPLRGAALAAALAGLVALEGSETTAYRDIAGVPT---
Era103 Lyz103 MSVKKALAGGACSLALVTASFFGIVTDKVRISQEGLEHLIDCEGCKRQAYKDGAGVPT---
T4 E MNIFEMNRIDERLRLKIYKDTEGYYT---
Sequence alignment of the N-terminus of phage KBNP1315, P1, 21, phiKMV, Era103 and T4 endolysin.
TMDs are indicated in gray-box. The negatively charged residues in N-region, acting as the anchor, are indicated in red. Catalytic triad are highlighted in yellow. An arrow indicates the cleavage site predicted by SignalP4.1 server.
