# Supplementary material for: Complete Genomic and Lysis-Cassette Characterization of the Novel Phage, KBNP1315, which Infects Avian Pathogenic Escherichia coli (APEC)
Source: PLoS One. 2015 Nov 10;10(11):e0142504. doi: 10.1371/journal.pone.0142504 (PMC4640515; doi:10.1371/journal.pone.0142504)
Supplement: S3 Fig — (PPTX) [file pone.0142504.s003.pptx]

## Slide 1
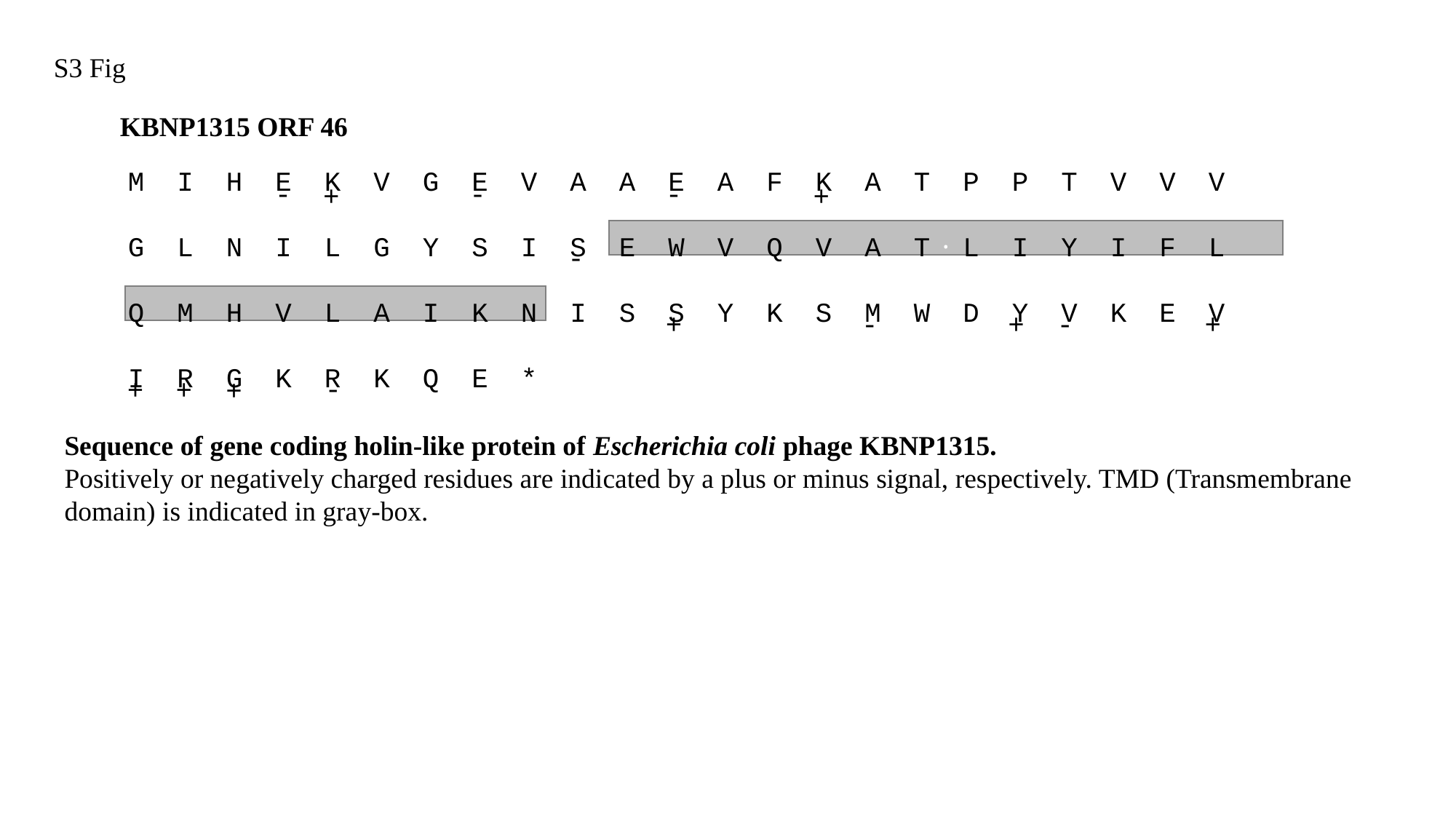

S3 Fig
KBNP1315 ORF 46
M I H E K V G E V A A E A F K A T P P T V V V G L N I L G Y S I S E W V Q V A T L I Y I F L Q M H V L A I K N I S S Y K S M W D Y V K E V I R G K R K Q E *
-
-
-
+
+
.
-
+
-
+
-
+
-
+
+
+
Sequence of gene coding holin-like protein of Escherichia coli phage KBNP1315.
Positively or negatively charged residues are indicated by a plus or minus signal, respectively. TMD (Transmembrane domain) is indicated in gray-box.
